# Interventional Pain Management According to Evidence-Based Medicine

**DOI:** 10.5812/aapm.4514

**Published:** 2012-04-01

**Authors:** Farnad Imani, Poupak Rahimzadeh

**Affiliations:** 1 Department of Anesthesiology and Pain Medicine, Tehran University of Medical Sciences (TUMS), Tehran, Iran

**Keywords:** Pain Management, Evidence-Based Medicine, Pain

Evidence-based medicine (EBM) endeavors to apply the best available evidence gained from scientific methods to clinical decision making (1). It aims to assess the strength of the evidence based on both the risks and benefits of treatments and diagnostic tests. The quality of the evidence can be evaluated from the source type (mostly from meta-analyses and systematic reviews of double-blind, placebo-controlled clinical trials), as well as other factors which include statistical validity, efficacy, clinical relevance, currency, and peer-review acceptance (2). A systematic review is, however, the best method in order to identify, and critically evaluate all relevant research on the effectiveness of a particular treatment. Initially, EBM was called, “a critical appraisal,” as it described the application of basic rules of evidence. This evidence was first presented by a group of clinical epidemiologists at McMaster University in 1990, usage of this technique later expanded to all medical fields, and it has now found global acceptance. 

In our practice, it is generally accepted that interventional pain management techniques have gained a definite place in the management of chronic pain syndromes. Actually, the most important goal of pain medicine is to use a specific treatment; conservative and/or interventional, for the right patient at the right time. Therefore, treatment selection should be made according to the clinical diagnoses. In reality, patients receive treatments that vary both due to their geographical location, as well as the specialty of the treating physicians. According to the literature, the treatment of pain syndromes should involve a multidisciplinary approach and should ideally entail the evaluation and treatment of the patient by; physicians, physical therapists, and psychologists well-versed in the complex biopsychosocial and pathophysiological causes in the development and maintenance of pain syndromes. For the correct application of interventional pain management techniques, both a good theoretical knowledge, as well as practical experience is mandatory.

In evaluating the literature and developing recommendations, the Cochrane Database and other recent systematic reviews are emphasized the most. Efficacy of a procedure or drug is considered to have been demonstrated if the results of a randomized clinical trial (RCT) are found to give statistically significant greater pain reduction, versus a placebo for the primary outcome measure, and the results are then assessed by the centers responsible for levels of EBM. All medications or procedures with efficacy supported by at least one systematic review or positive placebo-controlled or procedure or dose-response RCT, in which the reduction of chronic pain is a primary or co-primary endpoint, are considered for inclusion. Published data, unpublished data (if available), and the clinical experience of the authors are used to evaluate each of these modalities in terms of their degree of efficacy, safety, tolerability, drug interactions, ease of use, and impact on health-related quality of life. Nowadays, with such a plethora of pain knowledge findings, the efficacy of pain management techniques have been described in multiple randomized controlled trials, observational studies, retrospective studies, and case reports. So, usually there is lots of existing information and data to support any clinical practice.

Finally, evidence-based practice guidelines are written by the organizations responsible, and these provide a good review of the literature in a context that makes it accessible and useful to both the clinician and researcher (3, 4). Having looked at this issue from different aspects, one comes to understand that in the new and modern world of pain practice, EBM, systematic reviews, and guidelines are a major part of interventional pain management. A well designed management strategy starts with an accurate evaluation process to identify the pain diagnosis. It is of the utmost importance that so-called red flags are checked first, as they may be indicative of an underlying primary pathology, which needs adequate attention and treatment prior to the application of symptomatic pain management techniques. With interventional pain management techniques, a non-algorithmic approach to patients can be problematic or overly expensive, so interventionist should always remain cautious. 

Consequently, evidence based practice guidelines are of greater practical value when they are specific for each different pain diagnosis. It is recommended that the interventionist takes note of the algorithmic pattern and follows the rules, meanwhile observing the patient for potential red flags.

The series of articles published in the EBM section of pain practice and pain physician journals have covered the most important pain diagnoses and using these guidelines is strongly recommended to all pain physicians. These guidelines could help to solve the above mentioned impediments. These articles have been published between 2009 up to the present time. Different pain syndromes such as; trigeminal neuralgia, cervical and lumbar radicular pain, facetogenic pain, headaches, phantom pain, and post herpetic neuralgia, have been described in these articles and an algorithmic treatment approach has been planned for them. Essentially, this series of articles forms global guidelines for interventional pain management.

Due to the continual development of more specific diagnostic tools and to the improved understanding of pathophysiology, and consequently the mechanism of action of the different pain treatment options, it is generally accepted that treatment selection for chronic pain syndromes will become based more on the mechanism. Careful attention to this evolution is warranted and, when necessary, updates to the guidelines should be made. More and more guidelines are being released according to the recent literature and if necessary these are corrected by the latest findings (5, 6). Based on the philosophy that guideline panels should make recommendations on whether to administer, or not administer, a particular intervention, the taskforce chose to classify recommendations into strong and weak levels. The relationship between the quality of evidence and strength of the recommendation are complex issues, which requires the careful consideration of numerous factors. For this purpose multiple meetings and panels have been facilitated by pain organizations to gather different opinions in order to design or revise a guideline (6, 7).

The modern pain physician realizes that scientific and relevant evidence is essential in clinic care, policy-making, dispute resolution, and law. Thus, evidence-based pain practice provides strong, acceptable, trustworthy information by; systematically acquiring, analyzing and transferring research findings into clinical, management, and policy arenas (7-9). It is hoped that in the near future more attention will be payed to these aspects of pain practice by pain physicians and that further useful guidelines for each parts of this field are created, so a treatment can only be recommended when the effects of it, have been proven in well-designed trials and analyzed by centers with appropriate expertise.
